# From Dysplasia to Carcinoma: Expression Patterns of Dermokine, Matriptase, and Tryptase in OPMD

**DOI:** 10.1111/odi.70043

**Published:** 2025-07-24

**Authors:** Lara Maria Alencar Ramos Innocentini, Mateus Gonçalves Miranda, Carol Kobori da Fonseca, Pedro Franco Ferreira, Leandro Dorigan de Macedo, Graziela Cavalcanti, Luiz Carlos Conti de Freitas, Ana Carolina Fragoso Motta, Alan Grupioni Lourenço, Katiuchia Uzzun Sales

**Affiliations:** ^1^ Department of Stomatology, Public Health and Forensic Dentistry, School of Dentistry of Ribeirão Preto University of São Paulo Ribeirão Preto SP Brazil; ^2^ Department of Cell and Molecular Biology and Pathogenic Bioagents, Ribeirao Preto Medical School University of São Paulo Ribeirão Preto SP Brazil; ^3^ Dentistry and Stomatology Division, Department of Ophthalmology, Otolaryngology, Head and Neck Surgery, Ribeirão Preto Clinical Hospital, Ribeirão Preto School of Medicine São Paulo University Ribeirão Preto SP Brazil; ^4^ Department of Ophthalmology, Otolaryngology, Head and Neck Surgery, Ribeirão Preto Clinical Hospital, Ribeirão Preto School of Medicine São Paulo University Ribeirão Preto SP Brazil; ^5^ Department of Basic and Oral Biology, Ribeirão Preto School of Dentistry University of São Paulo Ribeirão Preto SP Brazil

**Keywords:** dermokine, matriptase, oral dysplasia, oral squamous cell carcinoma, serine proteases, tryptase

## Abstract

**Background:**

The malignant transformation of oral potentially malignant disorders (OPMDs) lacks reliable molecular markers. Dermokine and matriptase are involved in epithelial differentiation and inflammation, while tryptase is associated with the tumor microenvironment, which may contribute to carcinogenesis. This study investigated these molecules as potential biomarkers for predicting the malignant progression of OPMDs.

**Methods:**

This was a cross‐sectional comparative study. Paired normal oral mucosa (NM) and OPMD tissues (*n* = 21) were assessed, while oral and oropharyngeal squamous cell carcinoma (OOSCC) samples (*n* = 64) were evaluated using tissue microarrays. Proteomic analysis of five OPMD cases identified dermokine, tryptase, and matriptase as potential biomarkers, further examined through clinical, histopathological, and immunohistochemical analyses across NM, OPMD, and OOSCC tissues.

**Results:**

Females comprised 52% of the OPMD group, whereas males accounted for 87.5% of OOSCC cases. Dermokine, tryptase, and matriptase showed higher expression in OPMD and OOSCC than NM. Dermokine was predominantly observed in OPMD, well‐differentiated OOSCC, and NM from patients who progressed to malignancy (*p* = 0.019). Matriptase expression shifted from membranous in NM to diffuse in OPMD and OOSCC, similarly to tryptase.

**Conclusion:**

Dermokine may represent an early marker of transformation, while altered matriptase patterns could help differentiate dysplasia from carcinoma.

## 1 Introduction

Oral potentially malignant disorders (OPMDs) have a global prevalence of 4.47%, with the highest rates observed in Asia (10.54%) and South America (18.55%) (Warnakulasuriya et al. 2021). These disorders represent a diverse group of lesions with varying histopathological features, among which the degree of epithelial dysplasia remains the most significant indicator of malignant potential. Reported rates of malignant transformation range from 1.4% to 49.5%, depending on the population studied (Iocca et al. 2020). Oral leukoplakia is the most common OPMD and is strongly associated with an increased risk of progression to malignancy (van der Waal et al. 1997; Wils and Brakenhoff 2025). While dysplasia grading is a relatively reliable prognostic tool, it is limited by subjectivity and considerable intra‐ and interobserver variability (Tilakaratne et al. 2019). Moreover, the etiology and biological behavior of OPMDs remain incompletely understood, despite their clinical relevance being closely tied to the risk of malignant transformation (Pindborg et al. 1997; Warnakulasuriya et al. 2021). These disorders also share established risk factors with oral cavity carcinomas, particularly tobacco and alcohol use. In contrast, oropharyngeal lesions are more frequently associated with Human Papillomavirus (HPV) infection (Lim and D’Silva 2024; Tan et al. 2023). Squamous cell carcinoma (SCC) is the most common histological type of cancer in these regions. It is often diagnosed at advanced stages, especially in developing countries, leading to poor outcomes and relatively low 5‐year survival rates (Bray et al. 2024; Chi et al. 2015). In Brazil, for instance, the 5‐year survival rates are reported to be 49% for oral cancer and 31% for oropharyngeal cancer (International Agency for Research on Cancer [IARC] 2023). Delays in seeking medical attention or referral to specialists for definitive diagnosis further contribute to late‐stage detection, significantly limiting the success of therapeutic interventions (Forastiere et al. 2001; Molinolo et al. 2009).

Without validated molecular biomarkers, histological grading remains the primary determinant of malignant potential in OPMDs Kumari et al. (2022). Therefore, identifying predictive biomarkers could enable more accurate risk stratification, closer monitoring of high‐risk cases, and more informed therapeutic decision‐making, ultimately improving early detection of oral cancer (Lorenzo‐Pouso et al. 2023; Bastías et al. 2024). In this context, the present study employed a discovery proteomics approach integrated with lesion diagnosis to identify prognostic signatures in patients with OPMDs. Protein profiles of contralateral normal oral mucosa (NM) and OPMD tissue were analyzed using laser microdissection (LM) to isolate target areas for evaluation. This strategy revealed several potential biomarkers, including dermokine, matriptase, and tryptase, which exhibited distinct expression patterns and may contribute to predicting the malignant transformation of OPMDs.

## 2 Materials and Methods

### 2.1 Study Design and Patients

This observational cross‐sectional study analyzed the expression of dermokine, matriptase, and tryptase in patients with (i) OPMDs and (ii) OOSCCs. Eligible patients were recruited at the Ribeirão Preto School of Dentistry (FORP) and Clinical Hospital of the Ribeirão Preto Medical School (HCFMRP), University of São Paulo (USP), Ribeirão Preto, Brazil, between 2017 and 2020. Adults aged ≥ 18 years with a diagnosis of OPMDs and OOSCCs were included. Patients with incomplete records or a prior history of tumors in the head and neck region were excluded. Clinical data collected included sex at birth (reported as biological sex), age, smoking status, alcohol consumption, and lesion site. Biopsy samples of contralateral NM and OPMDs (*n* = 21) were collected from patients. Also, OOSCC tissues (*n* = 85) were obtained from the Ribeirão Preto School of Dentistry (FORP). Tissue slides were subjected to hematoxylin and eosin (H.E.) staining and immunostaining. The study was approved by the local Institutional Review Boards (approval numbers: 56377916.1.0000.5440 and 50533515.6.0000.5440).

### 2.2 Oral Epithelial Dysplasia Grading

Histopathological diagnosis was published by the World Health Organization (WHO) in its 4th edition of the *Classification of Head and Neck Tumors* (El‐Naggar et al. 2017), which proposed a simplified binary grading system for OPMDs. This system categorizes dysplasia into two grades: low‐risk and high‐risk. In this classification, low‐risk dysplasia encompasses lesions with limited architectural disturbances and mild cytological atypia, often confined to the lower third of the epithelium. These changes pose a relatively lower risk of progression to OOSCC. In contrast, high‐risk dysplasia refers to lesions exhibiting more pronounced architectural disorganization, significant nuclear abnormalities, and involvement of more than two‐thirds of the epithelial thickness. These features are associated with a higher risk of malignant transformation and warrant more rigorous clinical management. This binary system was introduced to enhance interobserver agreement among pathologists and to facilitate clinical decision‐making by aligning histological findings with therapeutic strategies and follow‐up protocols.

### 2.3 Protein Extraction and Trypsin Digestion

Proteins were extracted and digested using a protocol involving 8 M urea to denature the samples. Reduction of proteins was performed with dithiothreitol (5 mM, 25 min at 56°C), followed by alkylation with iodoacetamide (14 mM, 30 min at room temperature, in the dark). To initiate digestion, the urea concentration was reduced to 1.6 M by dilution with 50 mM ammonium bicarbonate, and calcium chloride (1 mM) was added to the mixture. Trypsin digestion was carried out by incubating the samples with 2 μg of trypsin at 37°C for 16 h. The reaction was terminated using 0.4% formic acid. Peptides were then desalted using C18 stage tips, dried under vacuum, reconstituted in 0.1% formic acid, and stored at −20°C until further analysis via liquid chromatography coupled to tandem mass spectrometry (LC–MS/MS).

### 2.4 Mass Spectrometry

**FIGURE 1:**
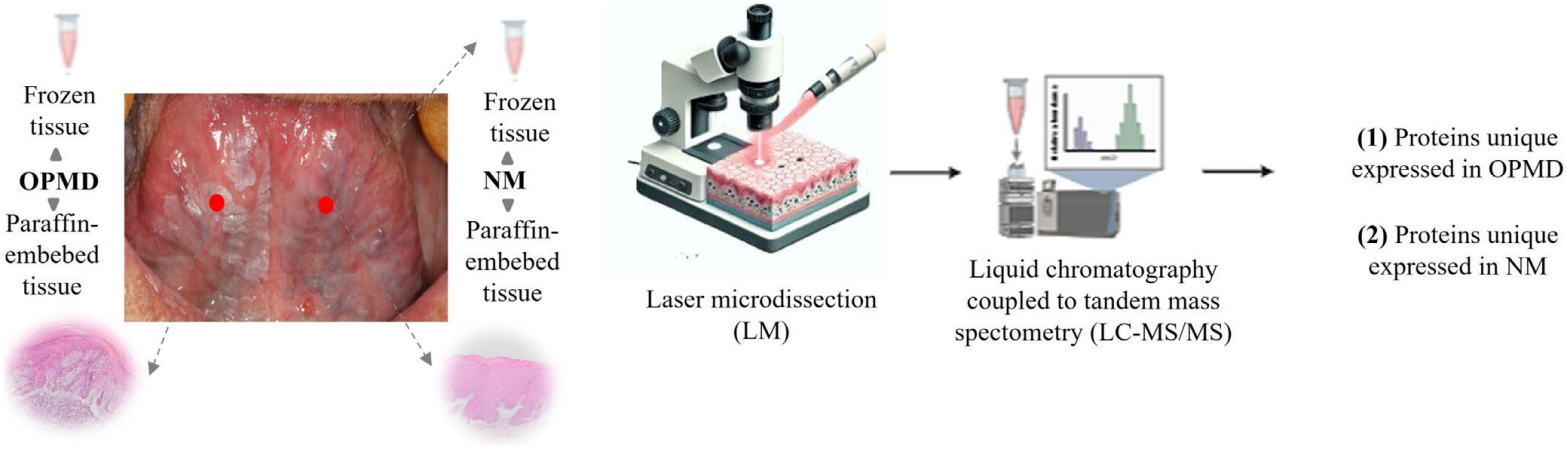
Protein analysis using OPMD group. Samples were obtained from oral potentially disorders (OPMDs) and contralateral normal tissue (NM) from the same patient (*n* = 5). Using histology‐guided laser capture microdissection (LM), we isolated the epithelial cells and analyzed them through mass spectrometry: (1) proteins exclusively expressed in OPMDs and (2) proteins exclusively expressed in NM (LM image source from bioart.niaid.nih.gov).

To identify pivotal markers involved in the malignant transformation of OPMDs into oral carcinomas, we performed a proteomic analysis on 10 epithelial samples obtained from five patients with oral tongue dysplasia, clinically presenting as leukoplakia. For each patient, paired samples were collected from the lesion site and the contralateral clinically normal mucosa. Frozen tissue sections were subjected to laser microdissection (LM), isolating only epithelial lining cells for protein extraction. Protein content was analyzed using label‐free quantitative mass spectrometry, and data were processed with Scaffold Q+ v.3.4.5, using parameters set to achieve a false discovery rate below 1%. Protein identification was based on normalized spectral counts (Aragão et al. 2012; Didangelos et al. 2011). This approach enabled us to map and compare dysplastic and normal mucosa's proteomic profiles within the same patient, identifying differentially expressed proteins spatially localized to OPMD and NM areas (Figure 1; Data S1).

### 2.5 Immunohistochemistry

For hematoxylin and eosin (H.E.) staining and immunohistochemistry (IHC), an expert oral pathologist (A.G.L.) previously analyzed all histomorphological features. The primary antibodies used were: Anti‐dermokine (concentration: 30 μg/mL—Cat.: E‐AB‐66152, produced in rabbit, Elabscience, Houston, Texas, USA); Anti‐matriptase (concentration: 20 μg/mL—Cat.: AF3946, produced in sheep, R&D Systems, Minneapolis, MN, USA); Anti‐tryptase (concentration: 20 μg/mL—Cat.: AF1667, produced in goat, R&D Systems, Minneapolis, MN, USA). All slides were scanned using the Olympus program OlyVIA. For the IHC technique, tissues were processed and embedded in paraffin blocks, and the blocks were sectioned into six μm‐thick slices and mounted onto salinized slides. Antigen retrieval was conducted in a microwave at pH 6.0 for 20 min at 10% power. The solutions employed included sodium citrate (0.01 M; pH: 6.0) for dermokine and tryptase and Tris‐EDTA (Tris 10 mM, EDTA 1 mM, Tween 20 0.05%, pH 9.0) for matriptase. Endogenous peroxidase blocking was performed with 3% hydrogen peroxide to reduce background staining. Primary antibodies were diluted in PBS with 1% BSA and incubated overnight at 4°C. Subsequently, samples were incubated at room temperature for 1 h with biotinylated secondary antibodies: Biotinylated anti‐rabbit IgG (H + L) BA‐1000 (Vector Laboratories, California, USA) for dermokine; Biotinylated anti‐sheep IgG (H + L) BA‐6000 (Vector Laboratories) for matriptase; and biotinylated anti‐goat IgG (H + L) BA‐9500 (Vector Laboratories) for tryptase. To visualize the staining, DAB (3,3′‐Diaminobenzidine; Sigma) was applied: 2 min for dermokine, 1 min 40 s for matriptase, and 3 min for tryptase. Counterstaining was performed with hematoxylin (for dermokine and matriptase) and toluidine blue 0.1% for tryptase. Slides were mounted with Permount (Thermo Fisher Scientific) for microscopic analysis. After analysis and selection, the slides were scanned using the Virtual Slide System (VS120/BX61VS—Olympus, Latin America Inc.) at 200× magnification.

### 2.6 Quantification of Staining

#### 2.6.1 OPMDs

All selected and scanned slides were analyzed using FIJI ImageJ software (version 1.54; WS Rasband, National Institute of Health, Bethesda, MD). The analysis employed the freehand tool to select the entire epithelial area, measured in μm^2^. The formula used for matriptase was Percentage of staining = (Total Stained Epithelial Area/Total Epithelial Area)*100, divided into membranous and diffuse staining. The formula applied for dermokine was Percentage of staining = (Total Stained Epithelial Area/Total Epithelial Area)*100. For tryptase, the formula used was Percentage of cells stained = (Total Stained cells/Total Epithelial Area)*100. Subsequently, the areas were classified as either positive or negative.

#### 2.6.2 OOSCCs

Analysis and quantification of dermokine, tryptase, and matriptase staining were conducted using QuPath, a scientific platform (version 0.4.4) (Bankhead et al. 2017). The region of interest (ROI) was automatically defined around the tissue in each TMA slide utilizing the tissue detection tools available in the software. An adjusted threshold was implemented to identify positively stained areas accurately. Measurements were automatically generated and modified as needed, classifying the areas as ‘Positive’ or ‘Negative’ to differentiate staining from the background. Data on stained areas were then calculated and exported for further quantitative analysis, allowing for a detailed assessment of the regions of interest in each TMA slide.

### 2.7 Bias

To minimize selection bias, paired samples of NM and OPMD were used, along with tissue microarrays (TMAs) for the OOSCC group. Blinded evaluators conducted histological and immunohistochemical assessments to reduce measurement bias. The study size was determined based on the availability of archived paired samples and tissue microarrays collected between 2017 and 2020.

### 2.8 Clinical and Histopathological Correlation

All clinical‐demographic information extracted from the medical and dental records database was correlated with antibody staining. The distinction between high and low expression was determined based on the overall mean expression observed in the samples analyzed (Alves et al. 2021).

### 2.9 Statistical Analysis

All data were analyzed using JAMOVI (Version 2.3). Nominal variables were assessed using Fisher's exact test, and continuous variables were analyzed using Student's *t*‐test. A one‐way ANOVA was used to assess whether dermokine expression varied significantly between lesion sites. Post hoc Tukey's tests were conducted to compare estimated marginal means between pairs of sites.

The Bonferroni correction was applied to control for Type I error when multiple comparisons were performed. Effect sizes were calculated using Cohen's *d*, with 95% confidence intervals reported. A significant level of 5% (*p* < 0.05) was adopted for all tests. Graphs were generated using GraphPad Prism 9.0 (GraphPad Software, San Diego, CA, USA).

## 3 Results

### 3.1 Sex Distribution Differs Between OPMD and OOSCC Groups, With Higher Male Prevalence in OOSCC

Table 1 presents data on sex at birth, tumor site, and histopathological classification for the OPMD and OOSCC groups. In the OPMD cohort, 52.3% of patients were female, whereas in the OOSCC group, 87.5% were male. The most frequently affected site in OPMD cases was the tongue (38.0%), while in OOSCC, the oropharynx was predominant (53.2%).

**TABLE 1:** Clinical and demographic features of OPMDs and OOSCCs.

Variables	OPMDs, *N* (%)	OOSCCs, *N* (%)
Total	21 (100)	64 (100)
Sex at birth		
Male	10 (47.7)	56 (87.5)
Female	11 (52.3)	8 (12.5)
Age (years)		
Median/SD	65 ± 6.48	60.5 ± 11.45
Clinical presentation		
Oral leukoplakia (homogeneous)	14 (66.6)	—
Oral leucoplakia (non‐homogeneous)	4 (19)	—
Proliferative verrucous leucoplakia	2 (9.52)	—
Oral lichenoid lesion	1 (4.7)	—
Site		
Tongue	8 (38)	30 (46.8)
Buccal mucosa	5 (24)	—
Alveolar edge	3 (14.4)	—
Hard palate	2 (9.2)	—
Gingiva	3 (14.4)	—
Oropharynx	—	34 (53.2)
Histopathological classification		
OPMDs—Low grade^a^	16 (76)	—
OPMDs—High grade^a^	5 (24)	—
OOSCCs—Well differentiated	—	27 (42.2)
OOSCCs—Moderate/Poorly differentiated	—	37 (57.8)

^a^ Grade of dysplasia (Binary system).

### 3.2 Spatial Proteomic Profiling of Tongue Lesions Uncovers Distinct Protein Signatures

**FIGURE 2:**
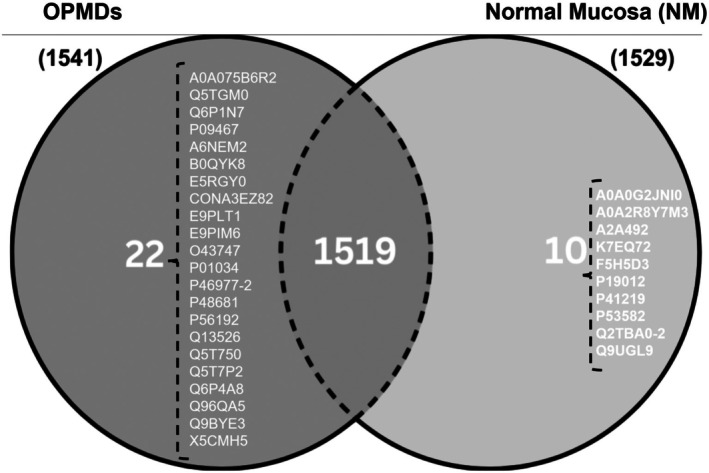
A total of 1561 proteins were observed in the samples, with 22 unique to OPMD samples and 10 unique to NM tissues.

A total of 1561 proteins were identified in the samples. Among them, 10 proteins were found exclusively in NM tissues, and 22 were found solely in OPMD lesions (Figure 2). Dermokine was detected exclusively in OPMD samples, while tryptase showed slightly elevated levels in lesions compared to normal mucosa. The proteomic profiling revealed these two proteins as differentially expressed between normal and pathological samples, suggesting their potential involvement in epithelial dysplasia and malignant transformation processes. Further immunohistochemical analyses were conducted to validate these findings and investigate their expression patterns across different histological grades.

### 3.3 Levels of Dermokine, Matriptase, and Tryptase Are Increased in OPMD

An in‐depth evaluation of dermokine, matriptase, and tryptase expression profiles revealed critical distinctions across various oral health and disease stages. Protein profiles were analyzed in the control group of NM and cases of low‐grade dysplasia (LGD) and high‐grade dysplasia (HGD). We also included well‐differentiated carcinoma (WDC) and moderately/poorly differentiated carcinoma (PDC) tissues.

**FIGURE 3:**
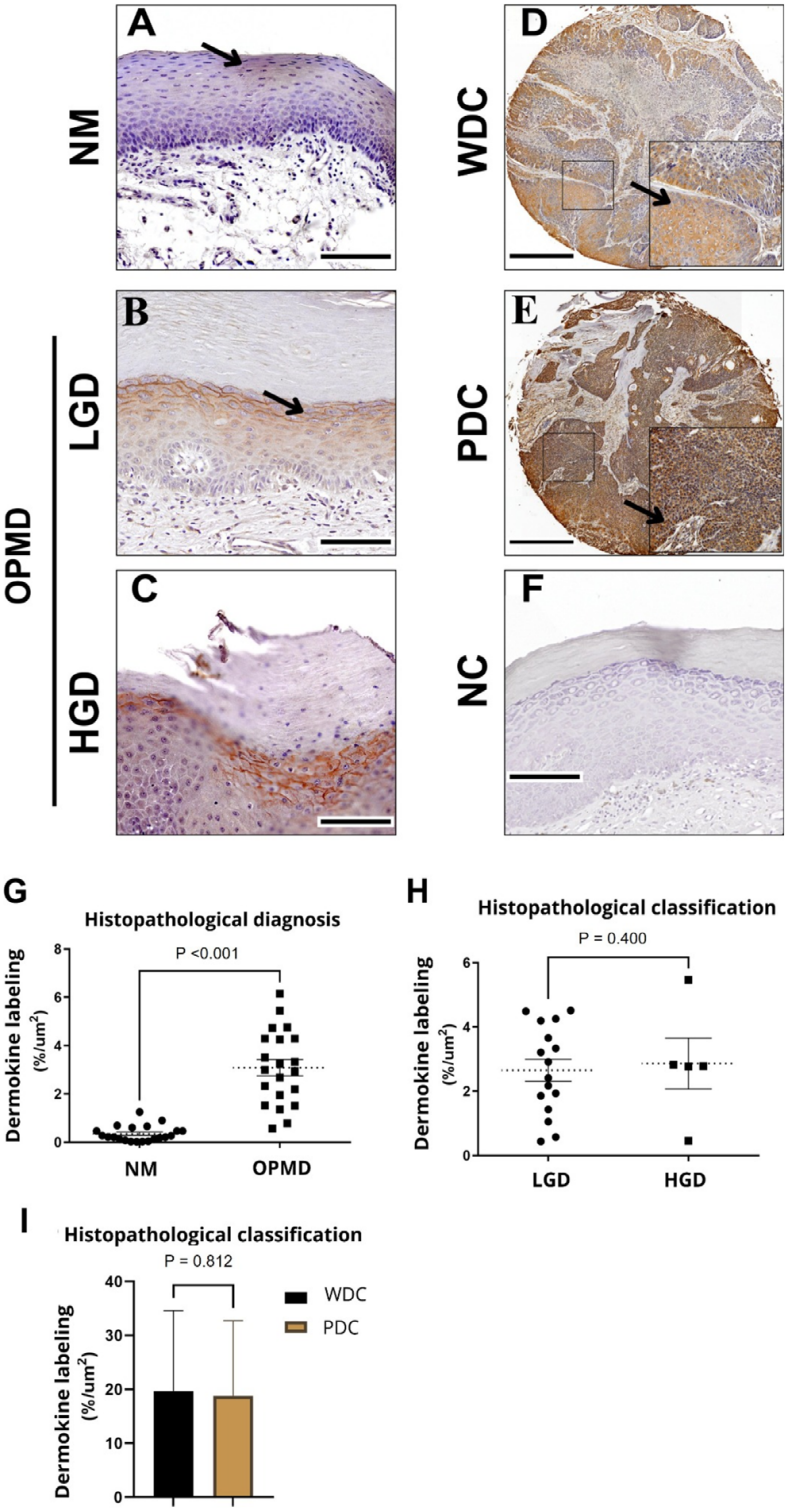
Dermokine presented higher levels in OPMD when compared with normal tissue. Dermokine staining in normal mucosa (NM) (A); in low‐grade dysplasia (LGD) (B); in high‐grade dysplasia (HGD) (C); well‐differentiated carcinoma (WDC) (D), and moderate/poorly differentiated carcinoma (PDC) (E). Negative controls using non‐immune rabbit IgG for dermokine (F). Black arrows indicate dermokine staining. Scale bars: A, B, C, F = 100 μm; D and E = 500 μm. Dermokine expression differs between NM and OPMD, *p* < 0.001, *t*‐test; with an effect size Cohen's *d* = 1.96, 95% CI, 1.2 to 2.73 (G). No statistical differences were found between LGD, HGD (H), WDC, and PDC (I).

**FIGURE 4:**
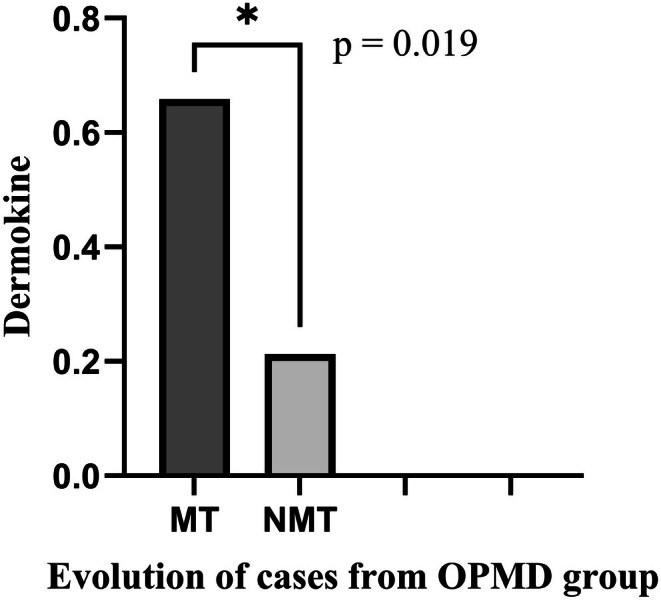
Dermokine presented higher levels in NM of patients that evolved to malignant transformation. Comparison of dermokine levels in clinically normal oral mucosa of cases with (MT, *n* = 3) and without (NMT, *n* = 17) malignant transformation (*p* = 0.019). Mann–Whitney *U*.

Immunohistochemistry findings indicated that clinically NM exhibited the lowest level of dermokine (0.34% of the stained area). In contrast, a notable increase in the percentage of epithelial area stained for dermokine was present in the OPMD group compared to the NM group (Figure 3G; *p* < 0.001, *t*‐test). However, there was no statistical difference between LGD and HGD. These values were significantly higher in WDC and PDC, with 19.68% and 18.81% of the stained area, respectively (Figure 3I). Interestingly, dermokine showed higher levels in the NM of patients who progressed to malignant transformation, *p* = 0.019 (Figure 4). No significant association was found between dermokine expression levels and the anatomical site of the lesions (Data S2). Although differences were observed in distribution, such as higher dermokine expression in lesions of the tongue and buccal mucosa, and lower expression in gingival lesions, these differences did not reach statistical significance.

**FIGURE 5:**
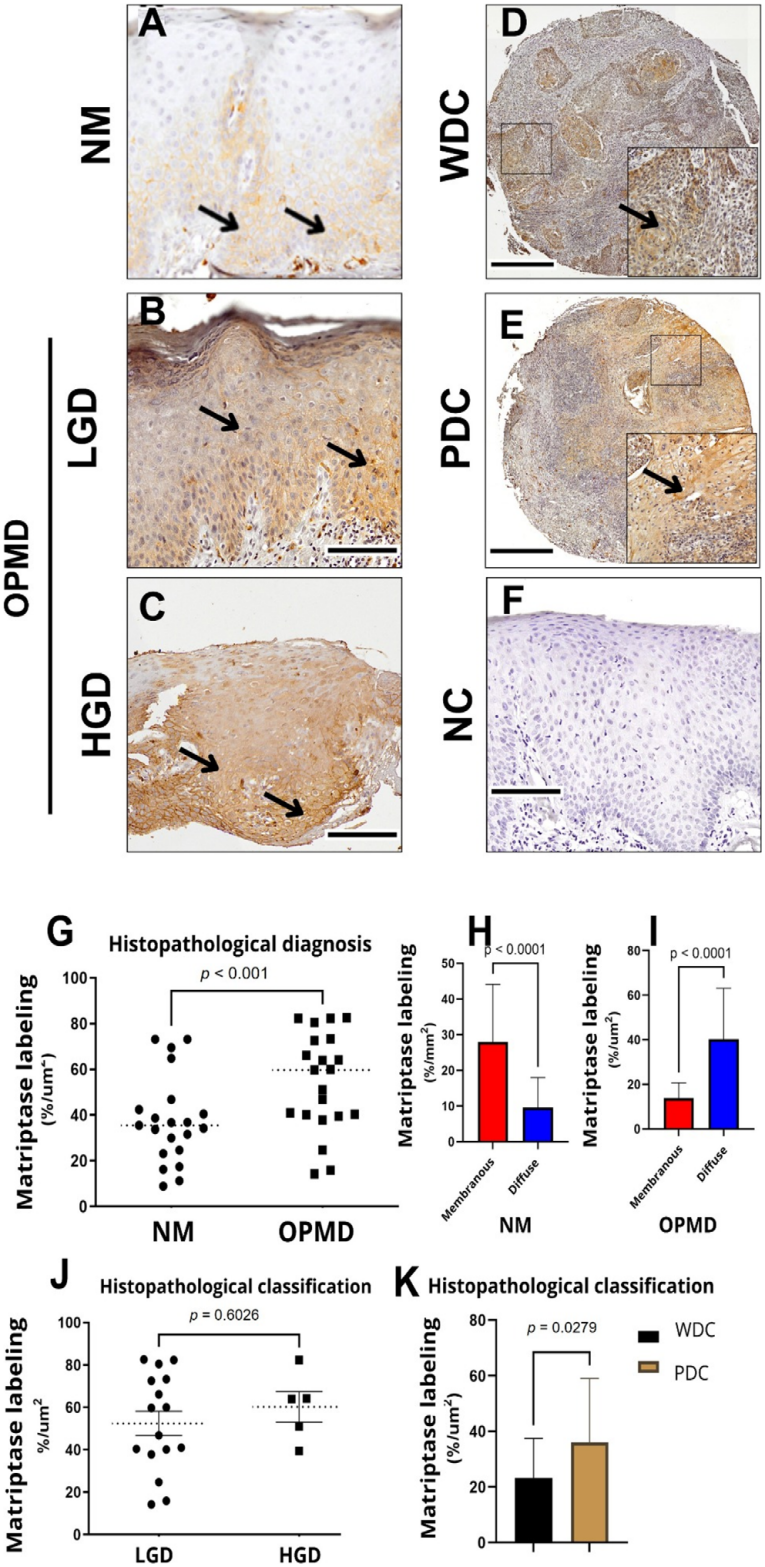
Matriptase presents a higher level in OPMDs and PDC, predominantly in its diffuse distribution. Matriptase staining in normal mucosa (NM) (A); in low‐grade dysplasia (LGD) (B); in high‐grade dysplasia (HGD) (C); well‐differentiated carcinoma (WDC) (D), and moderately/poorly differentiated carcinoma (PDC) (E). Negative controls using non‐immune sheep IgG for matriptase (F). Black arrows indicate matriptase staining. Matriptase differences in expression between NM and OPMD, *p* < 0.001, *t*‐test; with an effect size Cohen's *d* = 1.96, 95% CI, 1.2 to 2.73 (G). (H and I) Membrane and diffuse staining, *p* < 0.001, *t*‐test; with an effect size Cohen's *d* = 1.43, 95% CI, 0.672 to 2.17. Averages of 52.35% for low‐grade dysplasia (LGD) and 60.18% for high‐grade dysplasia (HGD), with no statistical differences were found between LGD, HGD (J), WDC, and PDC (K). Scale bars: A, B, C, E = 100 μm; D = 500 μm.

Matriptase quantification revealed an increase in the percentage of total epithelial area stained in biopsies with OPMD, with an average of 54.22%, compared to histologically normal biopsies (control group, NM), which had an average of 37.54% (Figure 5G, *p* < 0.001, *t*‐test). A significantly strong signal was observed at the plasma membrane in NM, averaging 27.99% of stained area, compared to OPMD, which averaged 9.58% (Figure 5H,I, *p* < 0.0001, *t*‐test). Conversely, diffuse staining was more pronounced in OPMD, with an average of 40.30% of stained area, compared to normal tissues, which showed an average of 13.90% (Figure 5I, *p* < 0.0001, *t*‐test). Figure 5J shows an average of 52.35% of the stained area for low‐grade dysplasia and 60.18% for high‐grade dysplasia, with no significant difference detected between these groups. The average amount of matriptase for well‐differentiated and moderately/poorly differentiated carcinomas was 23.28% and 36.00%, respectively, showing a significant difference between these groups (Figure 5K, *p* = 0.0279, *t*‐test). Thus, a considerable increase in diffuse matriptase was observed in dysplastic oral epithelium and moderately/poorly differentiated carcinomas.

**FIGURE 6:**
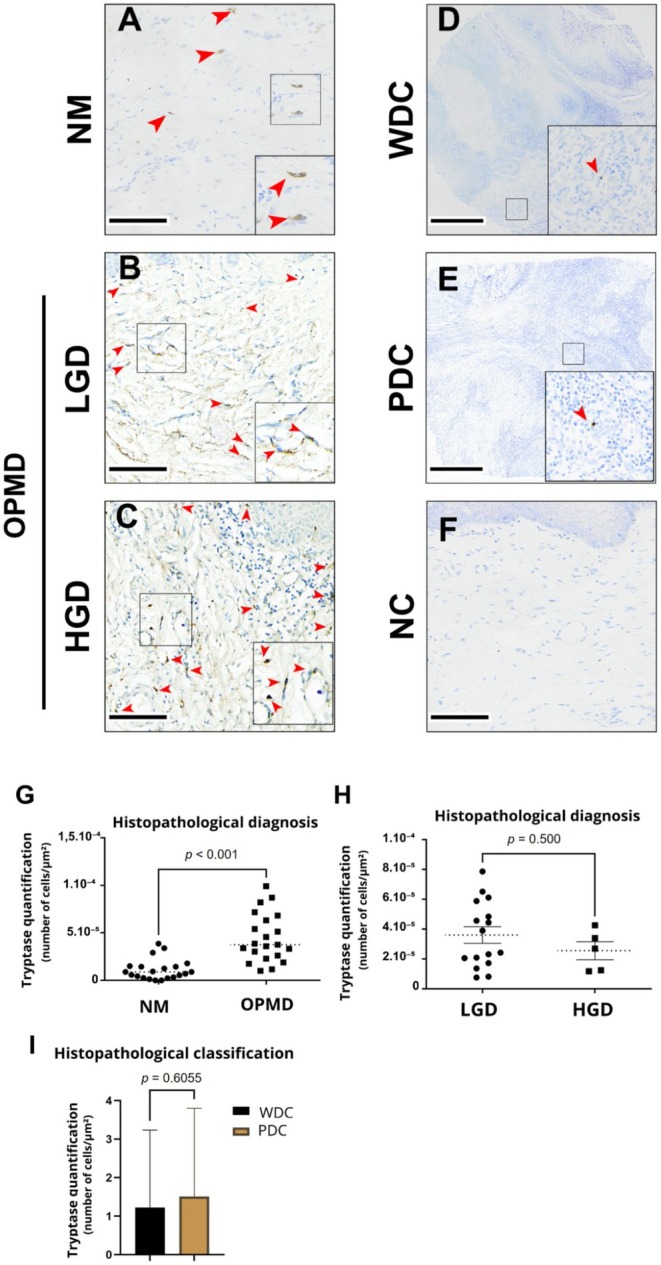
Tryptase presented higher levels in OPMDs. Tryptase staining in normal mucosa (NM) (A); in low‐grade dysplasia (LGD) (B); in high‐grade dysplasia (HGD) (C); well‐differentiated carcinoma (WDC) (D), and poorly differentiated carcinoma (PDC) (E). Negative controls using non‐immune goat IgG for tryptase (F). Red arrows indicate tryptase staining. Scale bars: A, B, C, F = 100 μm; D and E = 500 μm. Tryptase differences in expression between NM and OPMD, *p* < 0.001, *t*‐test; with an effect size Cohen's *d* = 1.71, 95% CI, 0.894 to 2.50 (G). No statistical differences were found between LGD, HGD (H), WDC, and PDC (I).

Tryptase was increased when OPMDs were analyzed through mass spectrometry and evaluated using LC–MS/MS, which prompted us to perform the tryptase immunohistochemical staining quantification. Interestingly, this analysis confirmed the mass spectrometry data. It was revealed as a cell count of 1.23·10^−5^/μm^2^ (SD 1.09·10^−5^) in normal mucosa and 4.47·10^−5^/μm^2^ (SD 2.55·10^−5^) in dysplastic mucosa (Figure 6G, *p* < 0.001, *t*‐test), showing an average of 3.6·10^−5^/μm^2^ (SD 2.55·10^−5^) in low‐grade dysplasia, and 2.55·10^−5^/μm^2^ in high‐grade dysplasia, with no significant difference detected between these groups (Figure 6H). Additionally, quantification showed a cell count of 1.22/μm^2^ (SD 2.01) in well‐differentiated carcinoma and 1.50/μm^2^ (SD 2.29) in moderate/poorly differentiated carcinoma (Figure 6I). A significant increase in the percentage of epithelial area stained for tryptase was observed in the OPMD group compared to the control group, as shown in Figure 6G (*p* < 0.001, *t*‐test). However, no significant differences were found between the LGD and HGD groups (Figure 6H, *p* = 0.500, *t*‐test), nor between the WDC and PDC groups (Figure 6I, *p* = 0.6055, *t*‐test).

## 4 Discussion

This study aimed to identify histopathological features and biomarkers associated with malignant progression in oral potentially malignant disorders (OPMDs). To achieve this, we selected tissue samples presenting varying degrees of epithelial dysplasia, representing precursor stages of oral carcinoma. We compared them with clinically normal oral mucosa from the same patients. Additionally, samples of well‐ and poorly differentiated oral squamous cell carcinomas (OSCCs) were included to assess biomarker expression across different stages of malignancy, given that the histopathological differentiation of OSCC is often correlated with patient prognosis (Agaimy and Weichert 2016). Due to mass spectrometry's complexity and technical demands, the proteomic discovery phase was conducted using a limited number of paired samples (*n* = 5), consistent with standard practice in exploratory biomarker research. Similar sample sizes have been employed in previous cancer biomarker studies, where the primary goal was to identify potential targets for further validation using IHC (Danda et al. 2018; Müller et al. 2018).

Dermokine has emerged as a potential biomarker in various types of cancer (Aiello and Kang 2019; Hasegawa et al. 2013; Uhland 2006; Utsunomiya et al. 2020). These previous findings align with our mass spectrometry results (Figure 2). These findings were confirmed with immunohistochemistry, showing that dermokine accumulates more in dysplastic lesions than in healthy tissues (Figure 3). This suggests a potential role for dermokine in progressing to oral epithelial dysplasia. Although dermokine expression could be influenced by the degree of keratinization of the mucosal site, our statistical analysis did not reveal significant associations between dermokine expression and anatomical location. Furthermore, the OOSCC cohort did not include samples from highly keratinized sites such as the palate and gingiva, minimizing the potential impact of keratinization‐related confounding in this group. Nevertheless, we acknowledge that subtle variations in keratinization at other oral sites could still have contributed to the observed expression patterns, and this warrants further investigation in future studies.

However, contrary to what has been reported by others (Hasegawa et al. 2013; Toulza et al. 2006), no significant differences were observed between the level of dermokine expression and various histological grades of dysplasia or SCC, nor with their clinical‐pathological presentation. We hypothesize that this unexpected result may be due to individual variability or sample size limitations. Interestingly, in our cohort, dermokine levels were three times elevated in the clinically normal mucosa (NM) of patients who later progressed to malignant transformation compared to patients who did not progress into malignant transformation (Figure 4). Indeed, this finding suggests that dermokine may have a broader role beyond keratinization, potentially contributing to early events in oral carcinogenesis. Importantly, the presence of dermokine in NM from patients who later progressed to malignancy could reflect either early field cancerization or a non‐specific inflammation‐related response. Nevertheless, in other epithelial diseases, such as psoriasis and congenital ichthyosis, inflammation typically arises due to barrier dysfunction caused by the absence of dermokine (Utsunomiya et al. 2020). In contrast, this manuscript shows that the increased presence of dermokine in normal mucosa from progressing patients likely reflects a distinct biological mechanism, potentially representing an early inflammatory response or epithelial stress within a pre‐malignant field, rather than a primary defect in epithelial barrier integrity.

Matriptase, a type II transmembrane serine protease of the MASP family, regulates physiological and pathological processes by cleaving cell surface substrates and is overexpressed in several cancers, including head and neck SCC, where it correlates with poor prognosis (List et al. 2006; Szabo and Bugge 2011; Uhland 2006; Murray et al. 2016). Immunohistochemical studies have demonstrated that matriptase is frequently elevated in carcinomas of various origins, reflecting a positive correlation with clinical and histological malignancy parameters such as tumor size, dysplasia degree, and tissue invasion (List et al. 2006; Uhland 2006; Sales, Friis, Abusleme, et al. 2015; Sales, Friis, Konkel, et al. 2015). Our study observed that matriptase protein expression patterns differ between NM and OPMDs. In normal tissue, the observed matriptase staining indicates that the protein is predominantly in the plasma membrane, while in lesions, the staining becomes diffused (Figure 5). Friis et al. (2013) demonstrated reciprocal activation of prostasin and matriptase zymogens at the plasma membrane. Upon activation, matriptase forms a complex with HAI‐1, leading to its rapid turnover and release into the extracellular environment. This may explain the reduced location of matriptase in the membrane in OPMDs, as it might be overactivated and released through a rapid turnover and thus be presented diffusely. No significant statistical differences were observed between the level of matriptase labeling and the various degrees of dysplasia, nor in their clinical‐pathological presentation. In SCC tissues, matriptase is more concentrated in poorly differentiated carcinomas than in well‐differentiated carcinoma samples. This may indicate that matriptase plays a role in carcinogenic progression. Additionally, low matriptase expressions were associated with oropharyngeal carcinomas, while high levels of protease are linked to oral carcinomas (Figure 5). This finding may correlate with oropharyngeal carcinomas' less aggressive behavior than oral carcinomas (Devaraja et al. 2020; Gale et al. 2017). Thus, low matriptase expression is associated with better‐differentiated grades of these lesions and with oropharyngeal carcinomas. Conversely, high levels of protease are associated with poorly differentiated histological grades and oral carcinomas.

Tryptase, the most abundant enzyme in mast cells, plays a key role in inflammation and angiogenesis, promoting cancer growth and progression in tissues such as the intestine, skin, and airways (Trivedi and Caughey 2010; Mohajeri et al. 2019; de Souza Junior et al. 2015; Ketabchi et al. 2007). Supporting these findings, tryptase expression was higher in dysplastic tissues than in normal mucosal tissue (Figure 6). Increased expression levels of matriptase accompanied tryptase expression levels in epithelial dysplasia. Matriptase is associated with the recruitment of dermal mast cells (da Silva et al. 2021; Sales, Friis, Konkel, et al. 2015), which may play a crucial role in tryptase levels (Cheng et al. 2007). However, no significant differences were observed between the levels of tryptase expression and the various histological grades of dysplasia or SCC, nor with their clinical‐pathological presentation.

## 5 Conclusion

The immunohistochemical signals of dermokine, matriptase, and tryptase increase in oral epithelial dysplastic lesions compared to normal tissue. From the clinical‐demographic correlations, higher matriptase expression is associated with less aggressive carcinomas, such as those of the oropharynx. These findings suggest that monitoring these biomarkers, particularly dermokine, may be valuable for the early detection of malignant transformation. Additional studies are needed to elucidate their specific roles and potential clinical applications.

## Author Contributions

**Lara Maria Alencar Ramos Innocentini:** conceptualization, investigation, writing – original draft, writing – review and editing, methodology, data curation. **Mateus Gonçalves Miranda:** conceptualization, investigation, writing – original draft, writing – review and editing, methodology, formal analysis, data curation. **Carol Kobori da Fonseca:** investigation, methodology, writing – review and editing, software, data curation. **Pedro Franco Ferreira:** methodology, conceptualization, investigation, writing – review and editing. **Leandro Dorigan de Macedo:** conceptualization, investigation, writing – review and editing, methodology. **Graziela Cavalcanti:** conceptualization, investigation, writing – review and editing, methodology. **Luiz Carlos Conti de Freitas:** conceptualization, investigation, methodology, writing – review and editing. **Ana Carolina Fragoso Motta:** conceptualization, investigation, funding acquisition, writing – review and editing, visualization, methodology, formal analysis, data curation, supervision, resources. **Alan Grupioni Lourenço:** conceptualization, investigation, methodology, validation, visualization, software, formal analysis, writing – review and editing. **Katiuchia Uzzun Sales:** conceptualization, investigation, funding acquisition, writing – review and editing, methodology, formal analysis, project administration, data curation, supervision.

## Ethics Statement

This study complies with the Declaration of Helsinki and has received the approval of the Research Ethics Committee of the Ribeirão Preto Dentistry and Medical Schools of the University of São Paulo. All participants were given and signed an informed consent.

## Conflicts of Interest

The authors declare no conflicts of interest.

## Supporting information


Data S1.

Data S2.

## Data Availability

The data that support the findings of this study are available from the corresponding author upon reasonable request.
