# Supplementary material for: From Dysplasia to Carcinoma: Expression Patterns of Dermokine, Matriptase, and Tryptase in OPMD
Source: Oral Dis. 2025 Jul 24;32(1):89–98. doi: 10.1111/odi.70043 (PMC13031407; doi:10.1111/odi.70043)
Supplement: Supplementary file 1 — Data S1. [file ODI-32-89-s002.docx]

| **Table 1. S1. Proteins represented in Venn diagram** | |
| --- | --- |
| **Proteins uniquely expressed in OPMD samples** | **Name and Functions** |
| A6NEM2;P51610-3;P51610-2;P51610;P51610-4 | Host cell factor C1 – regulation of immunity.  *Biological process – immune response* |
| A0A075B6R2 | Immunoglobulin heavy variable 4-4. V region of the variable domain of immunoglobulin heavy chains that participates in the antigen recognition (1).  *Biological process –* [*immunoglobulin mediated immune response*](https://www.ebi.ac.uk/QuickGO/term/GO:0016064) |
| Q6P1N7;C9JA35;A0A0G2JKZ1;A2AB90;A0A0G2JH37;A0A0A0MSV9;O15533-2;O15533;O15533-3 | TAP binding protein - Data suggest that tapasin (Tsn) binds to MHC I with suboptimal cargo and thereby adjusts the energy landscape in favor of MHC I complexes with immunodominant epitopes (2).  *Biological process – immune response* |
| Q96QA5;J3KRG2 | Gasdermin-A - This ultimately prevents bacterial penetration of the epithelial barrier and a subsequent systemic dissemination of the pathogen (9).  *Biological Process –* *Immune response* |
| X5CMH5 | ABC-type antigen peptide transporter - Catalytic activity. *Biological Process –* *adaptive immune* response |
| Q5TGM0;A0A087WY55;Q9NP79-2;Q9NP79 | Vesicle trafficking 1 - late endosome to vacuole transport via multivesicular body sorting pathway.  *Biological process – protein transport* |
| P09467;A0A3B3IUC7;Q5VZC3 | Fructose-1,6-bisphosphatase - Catalyzes the hydrolysis of fructose 1,6-bisphosphate to fructose 6-phosphate in the presence of divalent cations, acting as a rate-limiting enzyme in gluconeogenesis. Plays a role in regulating glucose sensing and insulin secretion of pancreatic beta-cells (5).  *Biological process – glucose regulation* |
| B0QYK8;E9PM95;J3KQL8;Q9BQE5 | Apolipoprotein L2 - lipoprotein metabolic process. GO:0042157  *Biological process - lipoprotein regulation* |
| E5RGY0;B4E1G1;Q9BUN8-2;Q9BUN8 | Derlin - Functional component of endoplasmic reticulum-associated degradation (ERAD) for misfolded lumenal proteins. May act by forming a channel that allows the retrotranslocation of misfolded proteins into the cytosol where they are ubiquitinated and degraded by the proteasome.  *Biological Process - response to stress* |
| CON__A3EZ82;Q6E0U4;Q6E0U4-4;C9JCN1;Q6E0U4-8;R4GMQ0;S4R3L7;C9J186;Q6E0U4-13;Q6E0U4-14;Q6E0U4-10;Q6E0U4-9;S4R2X7;M0R265 | Dermokine -May act as a soluble regulator of keratinocyte differentiation.  *Biological Process - cornified envelope assembly* |
| Q9BYE3;Q5TA76;Q5TA78;Q5TA79;Q5TA82;Q5TA81;O14633;Q5T7P3 | Late cornified envelope protein 3D - Precursors of the cornified envelope of the stratum corneum.  *Biological Process –* *keratinization* |
| Q5T750 | Protein KPLCE - epidermis development.  *Biological Process – involved in epidermis development.* |
| Q5T7P2;Q5T752;Q5TCM9;Q5T754;Q5T753;Q5T751 | Late cornified envelope protein 1A - Precursors of the cornified envelope of the stratum corneum.  *Biological Process –* *keratinization* |
| E9PLT1;E7EU05;P16671-4;P16671-3;P16671 | Platelet glycoprotein 4  *Biological Process - cell adhesion and lipid transport* |
| E9PIM6;E9PNQ8;P04216 | Thy-1 membrane glycoprotein, Thy-1 antigen - May play a role in cell-cell or cell-ligand interactions during synaptogenesis and other events in the brain.  *Biological Process - cell adhesion and regulation of cell migration.* |
| O43747;O43747-2;H3BR36;H3BUN9;H3BNR4 | Adaptor related protein complex 1 subunit gamma 1 – mainly intracellular protein transport.  *Biological Process - intracellular protein transport and vesicle-mediated transport.* |
| P01034 | Cystatin-C - As an inhibitor of cysteine proteinases, this protein is thought to serve an important physiological role as a local regulator of this enzyme activity.  *Biological Process – protease inhibitor* |
| P46977-2;P46977 | Dolichyl-diphosphooligosaccharide -protein glycosyltransferase subunit STT3A - Catalytic subunit of the oligosaccharyl transferase (OST) complex that catalyzes the initial transfer of a defined glycan (Glc_3_Man_9_GlcNAc_2_ in eukaryotes) from the lipid carrier dolichol-pyrophosphate to an asparagine residue within an Asn-X-Ser/Thr consensus motif in nascent polypeptide chains, the first step in protein N-glycosylation (5).  *Biological Process – post-translational protein modification* |
| P48681 | Nestin - CCR5 chemokine receptor binding.  *Biological Process – negative regulation of catalytic activity* |
| P56192 | Methionine--tRNA ligase, cytoplasmic -  Catalyzes the specific attachment of an amino acid to its cognate tRNA in a 2-step reaction: the amino acid (AA) is first activated by ATP to form AA-AMP and then transferred to the acceptor end of the tRNA (6).  *Biological Process –regulation of catalytic activity* |
| Q13526;K7EN45;K7EMU7;O15428 | Peptidyl-prolyl cis-trans isomerase - Catalytic activity.  *Biological Process –regulation of protein stability.* |
| Q6P4A8;F5H053 | Phospholipase B-like 1 - Exhibits a weak phospholipase activity, acting on various phospholipids, including phosphatidylcholine, phosphatidylinositol, phosphatidylethanolamine and lysophospholipids (8).  *Biological Process –* *phospholipid catabolic process* |

| **Table 2. S1. Proteins represented in Venn diagram** | |
| --- | --- |
| **Proteins uniquely expressed in clinically normal oral mucosa** | **Name and Functions** |
| A0A0G2JNI0;Q9UL52 | Transmembrane protease serine; Trypsin-like serine proteases 2  *Biological process –* [*proteolysis*](https://www.ebi.ac.uk/QuickGO/term/GO:0006508) |
| A0A2R8Y7M3;A0A2R8Y5S7;P35241;P35241-5;P35241-4;E9PQ82;E9PNV3;A0A2R8Y5P0;P35241-3;P35241-2 TMPRSS11E | Radixin - Probably plays a crucial role in the binding of the barbed end of actin filaments to the plasma membrane. |
| A2A492;Q7Z465-2;Q7Z465 | BCL2 interacting protein like |
| K7EQ72;K7ER02;B7Z7E1;P51911-2;P51911 | Calponin |
| F5H5D3;Q9BQE3;A0A1W2PQM2;F8VVB9;F8VRZ4;F8VS66;F8VX09;F8VWV9;A6NHL2-2;A6NHL2;F8VRK0;A0A7P0Z4A1;F8W0F6 | Tubulin alpha chain |
| P19012 | Tubulin is the major constituent of microtubules, a cylinder consisting of laterally associated linear protofilaments composed of alpha- and beta-tubulin heterodimers. Microtubules grow by the addition of GTP-tubulin dimers to the microtubule end, where a stabilizing cap forms. Below the cap, tubulin dimers are in GDP-bound state, owing to GTPase activity of alpha-tubulin. |
| P41219;P41219-2 | Peripherin - Class-III neuronal intermediate filament protein (By similarity). May form an independent structural network without the involvement of other neurofilaments or may cooperate with the neuronal intermediate filament proteins NEFL, NEFH, NEFM and INA to form a filamentous network (10). |
| P53582;H0Y9L0;D6RF24;H0Y903;H0Y955 | Methionine aminopeptidase 1  Cotranslationally removes the N-terminal methionine from nascent proteins. The N-terminal methionine is often cleaved when the second residue in the primary sequence is small and uncharged (Met-Ala-, Cys, Gly, Pro, Ser, Thr, or Val). Required for normal progression through the cell cycle. 1 and 2. |
| Q2TBA0-2;Q2TBA0 | Kelch-like protein 40 - Substrate-specific adapter of a BCR (BTB-CUL3-RBX1) E3 ubiquitin ligase complex that acts as a key regulator of skeletal muscle development (11). |
| Q9UGL9 | Cysteine-rich C-terminal protein 1 - epidermal differentiation complex (EDC) comprises a large number of genes that are of crucial importance for the maturation of the human epidermis. The newly identified EDC genes are likely to provide further insights into epidermal differentiation and they are potential candidates to be involved in skin diseases and carcinogenesis that are associated with this region of chromosome 1. |

| **Table 3. S1. Enriched biological process for differential expressed proteins** |  |  |  |
| --- | --- | --- | --- |
| **https://maayanlab.cloud/Enrichr/enrich#** |  |  |  |
| **Term** | **P-value** | **Adjusted P-value** | **Genes** |
| extracellular matrix organization (GO:0030198) | 2,22E-07 | 1,79E-04 | VTN;CDH1;CMA1;COL6A1;DPT;PLG;TGFBI;DCN;FBN1 |
| neutrophil degranulation (GO:0043312) | 1,08E-05 | 0,002233052 | HSPA8;TUBB;PSMD3;PSMC2;CPNE1;HLA-B;METTL7A;APEH;CTSB |
| neutrophil activation involved in immune response (GO:0002283) | 1,15E-05 | 0,002233052 | HSPA8;TUBB;PSMD3;PSMC2;CPNE1;HLA-B;METTL7A;APEH;CTSB |
| neutrophil mediated immunity (GO:0002446) | 1,21E-05 | 0,002233052 | HSPA8;TUBB;PSMD3;PSMC2;CPNE1;HLA-B;METTL7A;APEH;CTSB |
| regulation of complement activation (GO:0030449) | 1,42E-05 | 0,002233052 | VTN;CFH;CLU;CFB |
| regulation of immune effector process (GO:0002697) | 1,80E-05 | 0,002233052 | VTN;CFH;CLU;CFB |
| regulation of humoral immune response (GO:0002920) | 1,93E-05 | 0,002233052 | VTN;CFH;CLU;CFB |
| extracellular structure organization (GO:0043062) | 4,14E-05 | 0,003812589 | VTN;CDH1;COL6A1;TGFBI;DCN;FBN1 |
| external encapsulating structure organization (GO:0045229) | 4,25E-05 | 0,003812589 | VTN;CDH1;COL6A1;TGFBI;DCN;FBN1 |
| negative regulation of cell adhesion (GO:0007162) | 6,39E-05 | 0,004690903 | CDH1;PLG;TGFBI;KNG1 |
| antigen processing and presentation of exogenous peptide antigen via MHC class I, TAP-dependent (GO:0002479) | 6,39E-05 | 0,004690903 | PSMC6;PSMD3;PSMC2;HLA-B |
| regulation of G2/M transition of mitotic cell cycle (GO:0010389) | 7,77E-05 | 0,005145049 | PSMC6;TUBB;VPS4B;PSMD3;PSMC2 |
| antigen processing and presentation of exogenous peptide antigen via MHC class I (GO:0042590) | 8,28E-05 | 0,005145049 | PSMC6;PSMD3;PSMC2;HLA-B |
| positive regulation of proteolysis involved in cellular protein catabolic process (GO:1903052) | 1,03E-04 | 0,005964759 | PSMC6;PSMC2;CLU |
| negative regulation of mitotic cell cycle phase transition (GO:1901991) | 1,57E-04 | 0,007988327 | PSMC6;GPNMB;PSMD3;PSMC2 |
| interleukin-1-mediated signaling pathway (GO:0070498) | 1,71E-04 | 0,007988327 | IL1RN;PSMC6;PSMD3;PSMC2 |
| cellular protein complex disassembly (GO:0043624) | 1,78E-04 | 0,007988327 | VPS4B;APEH |
| positive regulation of RNA polymerase II transcription preinitiation complex assembly (GO:0045899) | 1,78E-04 | 0,007988327 | PSMC6;PSMC2 |
| proteolysis (GO:0006508) | 1,97E-04 | 0,008347971 | CPNE1;PLG;LAP3;APEH;CNDP2;CTSB |
| regulation of receptor-mediated endocytosis (GO:0048259) | 2,07E-04 | 0,008347971 | VTN;APOC3;CLU |
| negative regulation of blood coagulation (GO:0030195) | 2,23E-04 | 0,008577098 | VTN;PLG;KNG1 |
| negative regulation of cell-cell adhesion (GO:0022408) | 2,40E-04 | 0,008815112 | IL1RN;CDH1;PLG |
| cellular response to tumor necrosis factor (GO:0071356) | 2,67E-04 | 0,009379708 | PSMC6;DHX9;STAT1;PSMD3;PSMC2 |
| positive regulation of nucleocytoplasmic transport (GO:0046824) | 3,38E-04 | 0,011386967 | DHX9;CDH1;IPO5 |
| regulation of RNA polymerase II transcription preinitiation complex assembly (GO:0045898) | 3,79E-04 | 0,011881413 | PSMC6;PSMC2 |
| tumor necrosis factor-mediated signaling pathway (GO:0033209) | 3,82E-04 | 0,011881413 | PSMC6;STAT1;PSMD3;PSMC2 |
| cytokine-mediated signaling pathway (GO:0019221) | 4,53E-04 | 0,012896549 | HSPA8;IL1RN;PSMC6;STAT1;PSMD3;PSMC2;HLA-B;LMNB1 |
| regulation of cellular amine metabolic process (GO:0033238) | 4,59E-04 | 0,012896549 | PSMC6;PSMD3;PSMC2 |
| regulation of mRNA catabolic process (GO:0061013) | 4,63E-04 | 0,012896549 | HSPA8;PSMC6;PSMD3;PSMC2 |
| regulation of cellular amino acid metabolic process (GO:0006521) | 5,43E-04 | 0,013988862 | PSMC6;PSMD3;PSMC2 |
| regulation of proteasomal protein catabolic process (GO:0061136) | 5,43E-04 | 0,013988862 | USP14;PSMC6;PSMC2 |
| negative regulation of hemostasis (GO:1900047) | 5,54E-04 | 0,013988862 | VTN;KNG1 |
| negative regulation of cell cycle G2/M phase transition (GO:1902750) | 6,37E-04 | 0,015599658 | PSMC6;PSMD3;PSMC2 |
| negative regulation of coagulation (GO:0050819) | 7,61E-04 | 0,018084366 | VTN;KNG1 |
| regulation of cellular ketone metabolic process (GO:0010565) | 8,94E-04 | 0,019834367 | PSMC6;PSMD3;PSMC2 |
| pre-replicative complex assembly (GO:0036388) | 8,94E-04 | 0,019834367 | PSMC6;PSMD3;PSMC2 |
| regulation of mRNA stability (GO:0043488) | 9,08E-04 | 0,019834367 | HSPA8;PSMC6;PSMD3;PSMC2 |
| negative regulation of G2/M transition of mitotic cell cycle (GO:0010972) | 9,78E-04 | 0,020791672 | PSMC6;PSMD3;PSMC2 |
| reverse cholesterol transport (GO:0043691) | 0,001130831 | 0,022381089 | APOC3;CLU |
| cellular response to interleukin-1 (GO:0071347) | 0,00113406 | 0,022381089 | IL1RN;PSMC6;PSMD3;PSMC2 |
| protein deubiquitination (GO:0016579) | 0,001135674 | 0,022381089 | USP14;USP7;PSMC6;PSMD3;PSMC2 |
| regulation of hematopoietic stem cell differentiation (GO:1902036) | 0,001208636 | 0,023251855 | PSMC6;PSMD3;PSMC2 |
| protein modification by small protein removal (GO:0070646) | 0,001315336 | 0,023836928 | USP14;USP7;PSMC6;PSMD3;PSMC2 |
| negative regulation of cellular process (GO:0048523) | 0,001336345 | 0,023836928 | FGFBP1;GPNMB;VPS4B;PLG;TGFBI;CLU;KNG1 |
| NIK/NF-kappaB signaling (GO:0038061) | 0,001362253 | 0,023836928 | PSMC6;PSMD3;PSMC2 |
| cellular protein metabolic process (GO:0044267) | 0,001404898 | 0,023836928 | PLG;RPL10A;KNG1;UBE2L3;FARSB;FBN1 |
| regulation of hematopoietic progenitor cell differentiation (GO:1901532) | 0,001416055 | 0,023836928 | PSMC6;PSMD3;PSMC2 |
| regulation of transcription from RNA polymerase II promoter in response to hypoxia (GO:0061418) | 0,001416055 | 0,023836928 | PSMC6;PSMD3;PSMC2 |
| protein targeting to lysosome (GO:0006622) | 0,001570881 | 0,025385442 | HSPA8;CLU |
| positive regulation of interferon-alpha production (GO:0032727) | 0,001570881 | 0,025385442 | DHX9;STAT1 |
| negative regulation of cellular component organization (GO:0051129) | 0,001705085 | 0,027013891 | HSPA8;APOC3;CLU |
| anaphase-promoting complex-dependent catabolic process (GO:0031145) | 0,001961025 | 0,030471318 | PSMC6;PSMD3;PSMC2 |
| Wnt signaling pathway, planar cell polarity pathway (GO:0060071) | 0,002028537 | 0,030813906 | PSMC6;PSMD3;PSMC2 |
| positive regulation of transcription initiation from RNA polymerase II promoter (GO:0060261) | 0,0020799 | 0,030813906 | PSMC6;PSMC2 |
| positive regulation of proteasomal protein catabolic process (GO:1901800) | 0,002097481 | 0,030813906 | PSMC6;PSMC2;CLU |
| regulation of transcription from RNA polymerase II promoter in response to stress (GO:0043618) | 0,002167868 | 0,031279244 | PSMC6;PSMD3;PSMC2 |
| regulation of establishment of planar polarity (GO:0090175) | 0,002239709 | 0,031676528 | PSMC6;PSMD3;PSMC2 |
| regulation of mitotic cell cycle phase transition (GO:1901990) | 0,002299618 | 0,031676528 | PSMC6;TUBB;PSMD3;PSMC2 |
| SCF-dependent proteasomal ubiquitin-dependent protein catabolic process (GO:0031146) | 0,002313014 | 0,031676528 | PSMC6;PSMD3;PSMC2 |
| regulation of interferon-alpha production (GO:0032647) | 0,002456963 | 0,032638646 | DHX9;STAT1 |
| regulation of stem cell differentiation (GO:2000736) | 0,002464056 | 0,032638646 | PSMC6;PSMD3;PSMC2 |
| negative regulation of cell death (GO:0060548) | 0,002701853 | 0,035211242 | GPNMB;VPS4B;CLU |
| regulation of cell cycle phase transition (GO:1901987) | 0,002784153 | 0,035599074 | PSMC6;PSMD3;PSMC2 |
| regulation of blood coagulation (GO:0030193) | 0,002863787 | 0,035599074 | VTN;KNG1 |
| response to glucocorticoid (GO:0051384) | 0,002863787 | 0,035599074 | IL1RN;UBE2L3 |
| positive regulation of protein import into nucleus (GO:0042307) | 0,003078249 | 0,037685226 | CDH1;IPO5 |
| negative regulation of wound healing (GO:0061045) | 0,003300019 | 0,039797241 | VTN;KNG1 |
| post-translational protein modification (GO:0043687) | 0,003466005 | 0,04118429 | PSMC6;PSMD3;PSMC2;KNG1;FBN1 |
| positive regulation of protein import (GO:1904591) | 0,003529054 | 0,041325731 | CDH1;IPO5 |
| supramolecular fiber organization (GO:0097435) | 0,003729697 | 0,042850291 | TPM3;VPS4B;COL6A1;DPT;TGFBI |
| negative regulation of proteasomal ubiquitin-dependent protein catabolic process (GO:0032435) | 0,00376531 | 0,042850291 | USP14;USP7 |
| ubiquitin-dependent protein catabolic process (GO:0006511) | 0,003866822 | 0,043394338 | PSMC6;VPS4B;PSMD3;PSMC2;UBE2L3 |
| endodermal cell differentiation (GO:0035987) | 0,004008745 | 0,044370769 | VTN;COL6A1 |
| Fc-epsilon receptor signaling pathway (GO:0038095) | 0,004315634 | 0,047122054 | PSMC6;PSMD3;PSMC2 |
| Fc receptor signaling pathway (GO:0038093) | 0,004425205 | 0,047674211 | PSMC6;PSMD3;PSMC2 |
| sulfur compound biosynthetic process (GO:0044272) | 0,004536455 | 0,048229679 | GSTM3;DCN;CNDP2 |
| cellular response to lectin (GO:1990858) | 0,004764019 | 0,049350347 | PSMC6;PSMD3;PSMC2 |
| stimulatory C-type lectin receptor signaling pathway (GO:0002223) | 0,004764019 | 0,049350347 | PSMC6;PSMD3;PSMC2 |

**References**

1. Lefranc, M. P. (2014). Immunoglobulin and T cell receptor genes: IMGT® and the birth and rise of immunoinformatics. Frontiers in Immunology, 5, 22. doi:10.3389/fimmu.2014.00022
2. Fleischmann, G., Fisette, O., Thomas, C., Wieneke, R., Tumulka, F., Schneeweiss, C., Springer, S., Schäfer, L. V., & Tampé, R. (2015). Mechanistic basis for epitope proofreading in the peptide-loading complex. Journal of Immunology, 195(9), 4503–4513. doi:10.4049/jimmunol.1501515
3. Addlagatta, A., Hu, X., Liu, J. O., & Matthews, B. W. (2005). Structural basis for the functional differences between type I and type II human methionine aminopeptidases. Biochemistry, 44(45), 14741–14749. doi:10.1021/bi051691k
4. Hu, X., Addlagatta, A., Lu, J., Matthews, B. W., & Liu, J. O. (2006). Elucidation of the function of type 1 human methionine aminopeptidase during cell cycle progression. Proceedings of the National Academy of Sciences of the United States of America, 103(48), 18148–18153. doi:10.1073/pnas.0608389103
5. Ramírez, A. S., Kowal, J., & Locher, K. P. (2019). Cryo-electron microscopy structures of human oligosaccharyltransferase complexes OST-A and OST-B. Science, 366(6471), 1372–1375. doi:10.1126/science.aaz3505
6. Kaminska, M., Shalak, V., & Mirande, M. (2001). The appended C-domain of human methionyl-tRNA synthetase has a tRNA-sequestering function. Biochemistry, 40(47), 14309–14316. doi:10.1021/bi015670b
7. Kebede, M., Favaloro, J., Gunton, J. E., Laybutt, D. R., Shaw, M., Wong, N., Fam, B. C., Aston-Mourney, K., Rantzau, C., Zulli, A., Proietto, J., & Andrikopoulos, S. (2008). Fructose-1,6-bisphosphatase overexpression in pancreatic beta-cells results in reduced insulin secretion: A new mechanism for fat-induced impairment of beta-cell function. Diabetes, 57(7), 1887–1895. doi:10.2337/db07-1326
8. Xu, S., Zhao, L., Larsson, A., & Venge, P. (2009). The identification of a phospholipase B precursor in human neutrophils. FEBS Journal, 276(1), 175–186. doi:10.1111/j.1742-4658.2008.06771.x
9. Deng, W., Bai, Y., Deng, F., Pan, Y., Mei, S., Zheng, Z., Min, R., Wu, Z., Li, W., Miao, R., Zhang, Z., Kupper, T. S., Lieberman, J., & Liu, X. (2022). Streptococcal pyrogenic exotoxin B cleaves GSDMA and triggers pyroptosis. Nature, 602(7897), 496–502. doi:10.1038/s41586-021-04384-4
10. Gros-Louis, F., Larivière, R., Gowing, G., Laurent, S., Camu, W., Bouchard, J. P., Meininger, V., Rouleau, G. A., & Julien, J. P. (2004). A frameshift deletion in peripherin gene associated with amyotrophic lateral sclerosis. Journal of Biological Chemistry, 279(44), 45951–45956. doi:10.1074/jbc.M408139200
11. Ravenscroft, G., Miyatake, S., Lehtokari, V. L., Todd, E. J., Vornanen, P., Yau, K. S., Hayashi, Y. K., Miyake, N., Tsurusaki, Y., Doi, H., Saitsu, H., Osaka, H., Yamashita, S., Ohya, T., Sakamoto, Y., Koshimizu, E., Imamura, S., Yamashita, M., Ogata, K., Shiina, M., Bryson-Richardson, R. J., Vaz, R., Ceyhan, O., Brownstein, C. A., Swanson, L. C., Monnot, S., Romero, N. B., Amthor, H., Kresoje, N., Sivadorai, P., Kiraly-Borri, C., Haliloglu, G., Talim, B., Orhan, D., Kale, G., Charles, A. K., Fabian, V. A., Davis, M. R., Lammens, M., Sewry, C. A., Manzur, A., Muntoni, F., Clarke, N. F., North, K. N., Bertini, E., Nevo, Y., Willichowski, E., Silberg, I. E., Topaloglu, H., Beggs, A. H., Allcock, R. J., Nishino, I., Wallgren-Pettersson, C., Matsumoto, N., & Laing, N. G. (2013). Mutations in KLHL40 are a frequent cause of severe autosomal-recessive nemaline myopathy. American Journal of Human Genetics, 93(1), 6–18. doi:10.1016/j.ajhg.2013.05.004
