# Supplementary material for: From Dysplasia to Carcinoma: Expression Patterns of Dermokine, Matriptase, and Tryptase in OPMD
Source: Oral Dis. 2025 Jul 24;32(1):89–98. doi: 10.1111/odi.70043 (PMC13031407; doi:10.1111/odi.70043)
Supplement: Supplementary file 2 — Data S2. [file ODI-32-89-s001.docx]

**Association of Dermokine and Lesion Sites**

The data did not reveal significant correlations between dermokine expression and demographic characteristics such as sex and age. However, differences were observed in lesion sites, as well as smoking and alcohol consumption habits. The sex distribution was balanced between the groups, with a slight predominance of younger patients (0-65 years) in the low dermokine expression group. Regarding lesion sites, patients with high dermokine expression had a higher incidence of lesions on the tongue (41.7%) and buccal mucosa (33.3%), while gingival lesions were more common in the low-expression group (33.4%). Smoking was more prevalent in the high-expression group (25%) compared to the low-expression group (11.2%), and alcohol consumption was significantly more frequent among patients with high dermokine expression (58.4%) compared to those with low expression (22.3%). However, none of these correlations were statistically significant.

| **Table 1 S3. Clinical-Demographic Correlations of Dermokine in OPMDs** | | |
| --- | --- | --- |
|  | **Dermokine >2,70%** | **Dermokine <2,70%** |
| **Total cases** | **%** | **%** |
|  | N= (12) | N= (9) |
| **Histopathological Evaluation** |  |  |
| Low grade | 66 (8) | 88 (8) |
| High grade | 33 (4) | 12 (1) |
| **Sex at birth** |  |  |
| Female | 50 (6) | 55 (5) |
| Male | 50 (6) | 44 (4) |
| **Age** |  |  |
| 0-65 | 50 (6) | 55 (5) |
| ≥66 | 50 (6) | 44 (4) |
|  |  |  |
| **Site** |  |  |
| Tongue | 41.7 (5) | 22.2 (2) |
| Buccal mucosa | 33.3 (4) | 11.1 (1) |
| Alveolar edge | 16.6 (2) | 22.2 (2) |
| Hard palate | 8.4 (1) | 11.1 (1) |
| Gingiva | 0 (0) | 33.4 (3) |
| **Tobacco use** |  |  |
| Yes | 25 (3) | 11.2 (1) |
| No | 58.4 (7) | 44.4 (4) |
| ND | 16.6 (2) | 44.4 (4) |
| **Alcohol consumption** |  |  |
| Yes | 58.4 (7) | 22.3 (2) |
| No | 25 (3) | 33.3 (3) |
| ND | 16.6 (2) | 44.4 (4) |

ND -No Data

**Table 2. S3. ANOVA and post hoc comparisons of dermokine expression across lesion sites (normal mucosa)**

| Analysis | Comparison | Mean Difference | Standard Error | df | t | p-value (Tukey) |
| --- | --- | --- | --- | --- | --- | --- |
| ANOVA | — | — | — | — | 0.947 | 0.440 |
| Post hoc | Gingiva vs Tongue | 0.3087 | 0.544 | 17.0 | 0.5680 | 0.940 |
| Post hoc | Gingiva vs Buccal mucosa | -0.6049 | 0.609 | 17.0 | -0.9925 | 0.756 |
| Post hoc | Gingiva vs Palate | 0.3787 | 0.822 | 17.0 | 0.4608 | 0.967 |
| Post hoc | Tongue vs Buccal mucosa | -0.9137 | 0.574 | 17.0 | -1.5923 | 0.409 |
| Post hoc | Tongue vs Palate | 0.0700 | 0.796 | 17.0 | 0.0879 | 1.000 |
| Post hoc | Buccal mucosa vs Palate | 0.9837 | 0.842 | 17.0 | 1.1681 | 0.654 |

**Note.** The ANOVA result is shown in the first row; post hoc comparisons are based on estimated marginal. Post hoc Tukey’s tests.

**Table 3. S3. ANOVA and post hoc comparisons of dermokine expression across lesion sites (OPMDs)**

| Analysis | Comparison | Mean Difference | Standard Error | Df | t | p-value (Tukey) |
| --- | --- | --- | --- | --- | --- | --- |
| ANOVA | **—** | **—** | **—** | **—** | **1.26** | **0.321** |
| Post hoc | Gingiva vs Tongue | -0.372 | 0.817 | 17.0 | -0.456 | 0.968 |
| Post hoc | Gingiva vs Buccal mucosa | -1.300 | 0.915 | 17.0 | -1.420 | 0.504 |
| Post hoc | Gingiva vs Palate | 0.945 | 1.234 | 17.0 | 0.766 | 0.869 |
| Post hoc | Tongue vs Buccal mucosa | -0.928 | 0.862 | 17.0 | -1.077 | 0.708 |
| Post hoc | Tongue vs Palate | 1.318 | 1.195 | 17.0 | 1.103 | 0.693 |
| Post hoc | Buccal mucosa vs Palate | 2.246 | 1.265 | 17.0 | 1.775 | 0.318 |

**Note**. The ANOVA result is shown in the first row; post hoc comparisons are based on estimated marginal means. Post hoc Tukey’s tests.
